# A Review of Post-Operative Pancreatic Fistula Following Distal Pancreatectomy: Risk Factors, Consequences, and Mitigation Strategies

**DOI:** 10.3390/cancers17172741

**Published:** 2025-08-23

**Authors:** Jurgis Alvikas, Shakti Dahiya, Genia Dubrovsky

**Affiliations:** 1 Department of Surgery, University of Pittsburgh Medical Center, Pittsburgh, PA 15213, USA; 2 Surgical Services Division, VA Pittsburgh Healthcare System, Pittsburgh, PA 15240, USA

**Keywords:** distal pancreatectomy, post-operative pancreatic fistula (POPF), surgical outcomes, pancreatic cancer

## Abstract

Post-operative pancreatic fistula (POPF) is a serious yet far too common complication following distal pancreatectomy (DP), as it affects 20–30% of patients after DP. POPF raises the risk of other complications and causes delays to a patient’s oncologic care. In this review, we present the latest data on patient risk factors for developing POPF, such as obesity, smoking, young age, thick pancreas, lack of epidural anesthesia, hypoalbuminemia, and elevated drain amylase levels. Other risk factors that have been identified with irregular consistency include open surgical approach, non-malignant or neuroendocrine tumor pathology, simultaneous splenectomy, simultaneous vascular resection, and long operative time. We also review the consequences of POPF, which include hemorrhage, infection, delayed gastric emptying, re-operation, re-admission, delays in adjuvant chemotherapy initiation, reduced progression-free survival, and reduced overall survival. Finally, we present strategies that have been studied for avoiding POPF after DP, including reducing pressure at the sphincter of Oddi, the use of sealants and patches, optimizing pancreatic transection, strategies for post-operative drain placement, the use of post-operative somatostatin analogues, and pre-clinical studies of experimental devices and techniques that may inform future trials. This review informs readers on the current state of the art with regard to POPF after DP and sets the stage for future studies to improve patient outcomes.

## Simple Summary

Distal pancreatectomy (DP) is an operation frequently performed for tumors in the neck, body, and tail of the pancreas. One of the most dreaded complications after this surgery is a post-operative pancreatic fistula (POPF). POPF leads to significant patient morbidity and potentially mortality and has negative implications for a patient’s subsequent oncologic care. Thus, strategies to anticipate and avoid POPF after DP are distinctly needed. The aim of this review is to present the latest data to better understand which patients are at greatest risk for POPF, how POPF affects a patient’s post-operative clinical course, and what can be performed to avoid POPF. This review therefore provides clinicians and researchers with the background and context to improve patient care after DP and to help guide future studies to develop novel tools and techniques for prevention of POPF after DP.

## 1. Introduction

Distal pancreatectomy (DP) is an operation commonly performed for tumors within the left side of the pancreas, including the neck, body, and tail. Post-operative pancreatic fistula (POPF) is one of the most significant complications following DP. POPF occurs due to continued leakage of pancreatic fluid from the cut edge of the pancreas and has devastating and wide-ranging consequences for the patient, including hemorrhage and infection. Clinically relevant POPF (CR-POPF), which is defined as Grade B and C POPF by the 2016 ISGPS definition, leads to systemic symptoms such as organ failure and alters the clinical management of a patient following pancreatic resection [1]. This can include the need for additional medications such as antibiotics, additional procedures such as drain placement, and in the most extreme cases can result in death. Despite a variety of efforts and advancements in surgical technique and peri-operative management over the last several decades, the rate of CR-POPF after DP remains high at an estimated 20–30% [2].

Numerous studies have explored the topic of CR-POPF following DP, including risk factors for CR-POPF, clinical consequences of CR-POPF, and strategies for reducing CR-POPF. The conclusions from these research efforts have been heterogeneous, likely because of variations in patient populations, in pathologies, in technical details of the operations, and in levels of surgeon experience. Several systematic reviews and meta-analyses have sought to consolidate the existing data and summarize key conclusions. In this review, we provide the latest update on risk factors for developing CR-POPF following DP, describe the consequences for patients and hospital systems, and describe the state of current clinical and pre-clinical investigations on reducing CR-POPF.

## 2. Risk Factors for CR-POPF

CR-POPF can affect any patient following DP, but certain patients are at an increased risk. Numerous studies over the last several decades sought to identify the most reliable risk factors for CR-POPF (Table 1). A 2021 meta-analysis that included 43 studies and a total of 8864 patients found that smoking and an open surgical approach are associated with CR-POPF, while a history of diabetes mellitus was protective against it [3]. Of note, the authors found that elevated BMI was not a risk factor for CR-POPF on meta-analysis because of one seemingly outlier study by Watanabe et al. [4]. However, when reviewing the source data for their analysis, it is evident that there was a transcriptional error, as Watanabe et al. also show that BMI ≥ 25 is a risk factor for CR-POPF in their univariate analysis. Thus, on meta-analysis, BMI should also be listed as a significant risk factor for CR-POPF. The authors also compiled risk factors that were previously reported to be significantly associated with CR-POPF on multi-variate analysis. These include young age, low albumin, longer operating time, high BMI, non-pancreatic cancer, soft pancreas, thick pancreas, open approach, splenectomy, multi-visceral resection, vascular resection, and high post-operative drain amylase level [3]. However, the authors point out that there were inconsistencies in these particular risk factors, as some studies found no significance on evaluation, and others found a trend in the opposite direction. For example, several studies found that young age is a risk factor for CR-POPF, but a Japanese study instead found an increased rate of CR-POPF with patients older than 65 years [3,4]. It is important to note that the majority of studies included are small, heterogeneous retrospective studies that cannot adequately control for selection bias, patient and surgeon preference for surgical technique, or differences in patient populations. Given that well-designed studies comparing open to laparoscopic DP showed no differences in CR-POPF, the open surgical approach, splenectomy, and vascular resection may be surrogates for higher case complexity and not independent risk factors themselves. In fact, it is important to note that numerous studies of surgical approach have found that minimally invasive DP leads to lower blood loss and shorter hospital length of stay as compared to open DP, but the CR-POPF rates are equivalent.

A large 2019 international multi-institutional study by Ecker et al. analyzed 2026 patients that underwent DP and found that young age (<60 years), obesity, albumin < 3.5 g/dL, absence of epidural anesthesia, non-malignant or neuroendocrine pathology, concomitant splenectomy, and vascular resection are associated with CR-POPF [7]. Soft gland texture was significantly associated with CR-POPF on univariate analysis; however, on regression modeling, it was no longer an independent risk factor. Interestingly, other factors that were evaluated but found not to be significant included male sex, small duct size, intra-operative blood loss, the site of pancreatic transection, the method of pancreatic transection, and the operative approach (open, laparoscopic, or robotic). This study suffers from the same shortcomings as the systematic review described above, including its retrospective nature, insufficient detail to account for case complexity, and inadequate controls. Consistent with the finding that epidural anesthesia is protective against CR-POPF, a different single-center retrospective study by Kowalsky et al. identified higher peri-operative narcotic use to be associated with CR-POPF [10]. Pancreatic cancer pathology was protective in this study. Narcotic analgesic medications, such as morphine, can induce constriction of the sphincter of Oddi, thereby increasing pressure within the pancreatic duct and putting the cut edge of the pancreas at risk of leaking. This may also explain why epidural anesthesia and reduced need for IV narcotics are protective against CR-POPF.

Part of the difficulty in interpreting the literature is the change in the CR-POPF definition by ISGPS in 2016 [1]. What was formerly known as “grade A post-operative pancreatic fistula” was redefined as “biochemical leak” with no clinical significance and not a true post-operative pancreatic fistula. Prior to this change, studies often included patients with a “biochemical leak” and reported high POPF rates, some of which were without significant clinical consequences. For example, a meta-analysis from Peng et al. that only included studies prior to 2016 found that soft pancreatic texture, higher BMI, higher blood loss, need for transfusion, and longer operative time were all risk factors for CR-POPF [11]. These results should be interpreted with some caution given the change in CR-POPF definition. For example, a more recent meta-analysis looked specifically at pancreatic gland texture as a predictor of CR-POPF [12]. While CR-POPF was relatively higher in patients with a soft pancreatic texture, meta-analysis showed no significant difference. Another interesting single-center study investigated whether a serrated pancreatic contour and the ratio of the Hounsfield units of the pancreas to the surrounding visceral fat could be predictive of CR-POPF, since these factors correlate with a fatty gland [5]. These factors were not predictive, but the authors did show that certain pre-operative CT features, such as pancreatic thickness and elevated pancreatic Hounsfield units, are associated with CR-POPF. The authors conclude that in their series, a more robust gland that is thicker with less fat may be at higher risk for CR-POPF than an atrophied gland replaced by adipose tissue.

It is important to note that the risk factors for CR-POPF following DP vs. pancreaticoduodenectomy (PD) are different. Several risk factors are shared, including high BMI and high post-operative drain fluid amylase levels [13,14]. However, important differences also exist. For PD, a soft pancreatic gland and a small pancreatic duct are well-established risk factors for CR-POPF, while neoadjuvant therapy is a protective factor [13,15]. These features are not associated with CR-POPF as strongly in DP. For DP, unique factors such as pancreatic thickness and post-operative narcotic use influence the CR-POPF risk significantly [4,5,10]. One group hypothesized that CR-POPF rates may be different for DP and PD due to a difference in the density of acinar cells, and therefore a difference in the capacity to produce digestive enzymes, at different pancreatic transection locations [16]. This can be measured histologically and is termed the acinar score, with higher values leading to an elevated risk of CR-POPF.

Several risk calculators have been constructed to predict CR-POPF after DP. A study by Nassour et al. created a risk calculator using 692 distal pancreatectomy cases from the ACS-NSQIP database [9]. They found that young age, long operative time, drain fluid amylase on POD1, and the change in drain fluid amylase between POD1 and POD3 were the most important predictors for CR-POPF. The authors also provide a practical online calculator that can easily be used clinically by inputting these variables. Another study by De Pastena et al. constructed a calculator for both pre-operative and post-operative use, based on a large international dataset from the United States and the Netherlands [8]. The pre-operative calculator includes pancreatic neck thickness and pancreatic duct diameter as the only two variables. Thicker neck and larger duct diameter are associated with higher risk of CR-POPF. The intra-operative calculator also includes BMI, pancreatic texture, and operative time. The DISPAIR score, created using a cohort from Sweden and Finland, is also available for CR-POPF prediction following DP and includes transection site (neck vs. body/tail), pancreatic thickness, and presence of diabetes as important factors [17]. A thicker pancreas was associated with an increased risk of CR-POPF, while the presence of diabetes was protective. Transection at the neck actually carried a higher risk of CR-POPF, although this was mitigated by the fact that the neck was usually thinner, and this was a protective factor. It is important to note that these three risk calculators are meant to be used at different times during a patient’s care. While the risk calculator described by Nassour et al. can only be used post-operatively, the DISPAIR calculator is based on pre-operative factors that can be assessed via cross-sectional imaging. De Pastena et al. provide two risk calculators, and these are to be applied pre-operatively and post-operatively. A comparative study by Xu et al. sought to validate the De Pastena and DISPAIR calculators using a large Chinese cohort of 653 patients and found them to have similar discriminatory power [6]. While the performance of these risk calculators is promising, further large population-based validation studies that include multi-institutional cohorts will be necessary prior to their widespread implementation. In order to accomplish these studies, inclusion of additional variables on pancreatic thickness and texture will likely be required in national databases.

## 3. Consequences of CR-POPF

CR-POPF can have profound and long-lasting impacts for patients. CR-POPF is a major driver of a wide variety of post-operative complications, including hemorrhage, infection, delayed gastric emptying, re-operation, re-admission, hernia formation, and even death (Table 2) [18,19,20,21,22,23,24,25,26,27,28,29,30,31,32,33,34,35,36]. For patients with pancreatic cancer, development of CR-POPF after DP negatively impacts their oncologic outcomes. A meta-analysis by Grego et al. included both DP and PD cases and found that CR-POPF after pancreatic resection for pancreatic ductal adenocarcinoma is associated with higher local recurrence rates, shorter disease-free survival, and shorter overall survival [37]. Patients with CR-POPF were less likely to receive adjuvant therapy, and if they did, the time between surgery and adjuvant therapy initiation was longer [38,39,40]. CR-POPF has also been shown to dramatically increase cost. Several studies that focused on cost-effectiveness found that CR-POPF approximately doubles the healthcare costs following pancreatic resection [41,42]. One post-hoc analysis of a prospective randomized trial of pasireotide to prevent POPF found that cost in patients with CR-POPF increased significantly from a mean of $20,500 to $39,700 [41]. This study included both patients treated with PD and DP.

## 4. Techniques for Reducing CR-POPF

A variety of different strategies have been employed in an attempt to reduce CR-POPF after DP. These include pre-operative, intra-operative, and post-operative techniques and medications. A number of interesting and novel pre-clinical investigations have also attempted to reduce the risk of CR-POPF. Notable clinical and pre-clinical studies are summarized below.

### 4.1. Pre-Operative Strategies for Reducing CR-POPF

Several groups have investigated pre-operative strategies for reducing POPF after DP (Table 3). These have primarily focused on ways to reduce sphincter of Oddi tone and pressure, thereby promoting flow of pancreatic duct (PD) fluid into the duodenum and away from the cut edge of the pancreas. A prophylactic PD stent was first described in a small cohort study of 9 patients, where none of the patients developed POPF post-operatively [43]. A later cohort study compared POPF rates of 25 patients with a PD stent to 23 matched historic control patients who did not have stenting. All of the patients had at least 1 drain placed post-operatively. None of the patients in the stenting group developed POPF, while in the non-stent group, 2 patients developed grade A POPF and 3 patients developed CR-POPF [44]. Thus, stenting showed a significant reduction in POPF overall, although this study was limited by non-randomization. For example, the operative time was significantly lower in the stent group (205 min vs. 255 min), which is a known protective factor against POPF. Additionally, it is noteworthy that all stent patients underwent sphincterotomy, and 92% of patients had successful stent placement. One patient developed post-procedural pancreatitis which resolved within two days, and 5/23 patients (22%) had stent passage prior to planned removal at 8 weeks [44]. Thus, it is unclear if stent placement specifically was the critical intervention or rather sphincterotomy. One randomized controlled study has been performed to test the efficacy of prophylactic PD stenting before DP. In that study, 58 patients were randomized to either sphincterotomy and stent vs. no sphincterotomy or stent [45]. There was no significant difference in POPF or CR-POPF between the two groups (43% with stent vs. 22% without stent). However, it is important to note several limitations of this study. Two patients in the stent group did not have successful stent placement, and both of these patients developed CR-POPF grade B. Additionally, 6 patients (25%) experienced early passage of their stents prior to 3–4 weeks post-operatively. The study does not report whether any of these patients experienced CR-POPF. Finally, all stent patients were treated with sphincterotomy, and all study patients received octreotide. While octreotide has also been extensively studied to reduce POPF, it is not clear whether it would be beneficial in the setting of PD stenting. Stent occlusion is a common mode of stent failure, and this may be exacerbated if PD flow is reduced by octreotide [46,47]. A meta-analysis reviewed 4 studies of PD stenting following DP [48]. It did find a significant reduction in CR-POPF with stenting. However, this was driven by the results of three non-randomized studies, while the single randomized trial tended to favor the non-stent group [48]. Therefore, it is difficult to draw clear conclusions about the benefits of prophylactic PD stenting. Variables such as stent diameter and length and factors such as stent migration and occlusion may play a significant role in determining whether this intervention is beneficial or detrimental.

Another more recent strategy used to reduce pressure in the PD is Botox injection into the sphincter of Oddi. Notably, two German groups have published their results from small cohort studies. The initial study was a cohort study of 29 patients that underwent Botox injection, and 24 of these patients underwent DP [49]. None of the patients sustained CR-POPF, while a matched cohort of patients that underwent DP without Botox injection had a CR-POPF rate of 33%. A subsequent study with a similar design included 19 patients that underwent Botox injection followed by DP [50]. Here, the rate of CR-POPF was 32%, which was not significantly different compared to their matched historical control cohort that had a rate of 42%. The authors acknowledge the discrepancy between the two studies and provide several possible explanations. One factor to consider is the timing between Botox injection and subsequent surgical intervention. While the initial study had a median interval of 6 days, the later study had a median interval of 1 day. The optimal time interval to wait for the full effects of a Botox injection is not known but could be a relevant factor. Another aspect to consider is the location and depth of Botox injection. Although both studies aimed to perform the injections in the same manner, an inadequate depth of injection may not paralyze the sphincter muscle adequately and thus may limit therapeutic efficacy. Both of these studies have a limited sample size, and given the conflicting results, a larger randomized trial is planned and may provide more clarity [51].

### 4.2. Intra-Operative Strategies for Reducing POPF

Perhaps the most studied aspect of CR-POPF reduction has centered on intra-operative interventions and techniques. Specifically, studies have investigated autologous and synthetic patches for pancreatic stump coverage, techniques for pancreatic transection, and algorithms for drain management (Table 4). These various interventions will be reviewed here. Several well-designed randomized multicenter trials have used a fibrin sealant patch composed of collagen, fibrinogen, and thrombin (TachoSil) [52,53,54,55,56]. This patch is applied after pancreatic parenchymal transection and can be used with either suture ligation of the pancreatic stump or with stapler transection. Four randomized multicenter trials from Italy, France, South Korea, and the Netherlands have all concluded that the patch shows no benefit in reducing CR-POPF after DP [52,53,54,56]. A meta-analysis consisting of the trial from Italy and France, as well as two additional retrospective studies, also concluded that there is no benefit to this particular patch [55]. Interestingly, the Italian study did find a significant reduction in the drain amylase level on post-operative day 1 (3282 vs. 4703), even though the rate of grade A POPF was not different between groups [52]. This suggests that this patch may provide some early sealing benefit that is quickly overcome on subsequent post-operative days.

Another strategy is the use of a reinforced stapler to divide the pancreas. Importantly, there is significant variability in the materials available for stapler reinforcement, which include biodegradable materials such as polyglycolic acid (PGA) as well as biologic agents such as bovine pericardium and porcine intestine. Some notable examples include Seamguard (PGA-based), Neoveil (PGA-based), Peristrips Dry (bovine pericardium), and Biodesign staple line reinforcement (porcine intestinal submucosa). One of the first randomized controlled studies of a reinforced stapler came out of the United States and compared a stapler reinforced with either PGA or biologic tissue vs. a standard plain stapler [57]. They showed a significant reduction in CR-POPF (1.9% vs. 20%) with the use of a reinforced stapler. In this study, 57% of the reinforced group had a biologic mesh, and the rest had PGA. Of note, the patients in the reinforced group had a significantly lower BMI (27.9 vs. 31.0). Another study investigated PGA reinforcement exclusively, but the study protocol here involved suturing the PGA mesh after stapled pancreatic transection and also the use of fibrin glue to secure the mesh [58]. Again, there was a significant reduction in CR-POPF (11.4% vs. 28.3%). These encouraging results were tempered by several subsequent randomized studies. A multicenter randomized study from Japan used a PGA-reinforced stapler and showed no statistically significant reduction in CR-POPF (16.3% vs. 27.1%) [59]. However, since there was a trend towards a benefit in the reinforced stapler group, it is possible that this study of 119 patients was underpowered. Furthermore, the authors did note a significant benefit in patients with a pancreas < 14 mm thick at the point of transection (4.5% vs. 21.0%), indicating that the thickness may be an important factor to consider. Another multicenter randomized trial out of Sweden used exclusively biologic tissue for stapler reinforcement [60]. There was no benefit here in terms of CR-POPF (11% vs. 16%). Finally, a large multicenter randomized trial of 199 patients from France used exclusively PGA-reinforced staplers [61]. Here again, there was no reduction in CR-POPF (14% vs. 11%).

Several meta-analyses have tried to come to a consensus on PGA mesh and reinforced staplers [62,63,64]. These have included different randomized trials in their analyses, depending on the year of publication of the meta-analysis relative to the randomized trial. They have also included a handful of retrospective cohort studies that were not discussed here. When reviewing specifically the efficacy of a PGA mesh to reinforce the cut edge of the pancreas, there was a significant benefit for CR-POPF reduction [62]. Similarly, a meta-analysis reviewing reinforced staplers also concluded that there is a reduction in CR-POPF, although the studies included in this analysis used a wide variety of stapler reinforcements and thus should be interpreted with caution [63]. Furthermore, both of these meta-analyses were published prior to the most recent randomized trial from France that showed no benefit to a PGA-mesh-reinforced stapler. Thus the most recent meta-analysis that incorporates this French study concluded that there is no benefit to stapler reinforcement, although they notably excluded the original randomized trial from the United States in their analysis [64]. Altogether it is probably too early to draw a definitive conclusion, particularly because “stapler reinforcement” is such a broad category that includes a variety of devices. For example, the initial RCT from the United States used primarily a bovine pericardium to reinforce their staple line, while the more recent French study used exclusively synthetic PGA [57,61]. There were also some important differences in patient characteristics between the two studies. The US study had higher rates of open resection and lower rates of adenocarcinoma, and they excluded patients that had a pancreas too thick for the stapler. Of note, there is an ongoing randomized trial in Japan that will test the efficacy of a PGA mesh wrap after reinforced stapler transection of the pancreas [80].

Additional methods have also been studied to try to seal the pancreatic margin intra-operatively, with mixed results. A randomized study of pancreatic coverage with the falciform ligament showed no statistically significant reduction in CR-POPF (22% vs. 33%), but it did show fewer re-operations and re-admissions [65]. This seemed to indicate that the autologous tissue patch may be able to reduce the severity of CR-POPF. This type of patch reinforcement is further supported by a meta-analysis showing a reduction in CR-POPF with a variety of autologous patches, including falciform ligament, omental, and sero-muscular [66]. Additional non-autologous sealant patches to cover the pancreatic transection line have also been investigated. Simple application of cyanoacrylate glue in a single-arm pilot study was not convincing for strong efficacy [67]. A study from Korea compared a collagen-based thrombin-coated matrix with a thrombin-coated collagen patch [68]. In the small DP cohort of 25 patients, there was a significant reduction in CR-POPF (8.3% vs. 46.2%), although this did not translate to a difference in timing of drain removal, length of stay, re-admission, or mortality [68]. Furthermore, since the control group had a thrombin/collagen patch applied, it is difficult to draw conclusions without a “true” control. Other materials studied include the Hemopatch and Actiseal patch. The Hemopatch is made of bovine collagen coated with polyethylene glycol. The Actiseal patch is made of biodegradable polyurethane with an adhesive layer and a barrier layer. A randomized controlled trial of the Hemopatch did not show a significant difference in CR-POPF (16.3% vs. 23.2%), but it did show fewer overall complications [69]. A different single-arm study of the Hemopatch had a CR-POPF rate of 25% and thus also was not convincing for efficacy in its limited scope [70]. Similarly, a single-arm study of the Actiseal patch showed a CR-POPF rate of 17.5% and also requires further investigation in a randomized trial [71]. A recent meta-analysis of only randomized controlled trials aimed to review non-autologous modes of pancreatic staple line reinforcement [72]. A total of 9 studies were included, of which 4 were related to TachoSil. The rest included studies of PGA mesh, the Hemopatch, and the collagen-based thrombin-coated matrix. A subgroup analysis showed no benefit with the TachoSil patch, but a significant benefit of the other grouped reinforcement methods. These results were driven by the PGA mesh and the collagen/thrombin matrix [72]. Given the differences in these interventions, these amalgamated results should be interpreted with some caution.

As stapled parenchymal transection has become more frequently used during DP, different techniques with the stapler have also been studied. One consideration is the method of pancreatic compression with the stapler. Some surgeons recommend slow compression of the pancreas with the stapler over 5 min or more in order to flatten and thin out the parenchyma for better sealing. Conversely, others recommend rapid division of the parenchyma with the stapler to prevent the risk of ischemic injury from prolonged compression. There are no randomized studies, but retrospective studies are contradictory and complicated by non-randomization [73,74]. In future studies of CR-POPF, it will be important to report these specifics of pancreatic transection and to standardize intra-operative procedures to avoid possible confounding variables.

Prior to stapled transection of the pancreas, suture ligation was commonly performed. Several studies have tried to compare the two methods to see if there is a preferred means of dividing the PD. A small cohort study of 22 patients seemed to indicate that a combined method of stapling followed by suture ligation can reduce CR-POPF [75]. However, it is difficult to draw definite conclusions from such small non-randomized studies. Therefore, a large meta-analysis reviewed 37 studies and 5252 patients to determine if there was an optimal method of pancreatic remnant closure [76]. While total POPF was reduced with the use of a stapler (26.3% vs. 30.5%), CR-POPF was equivalent with stapling and suture ligation (14.2% vs. 17.4%). There was, however, a trend towards favoring the stapler. Other methods of pancreatic ligation were reviewed that included double closure with stapling and suturing combined, and this showed a benefit for total POPF with combined stapler and suture vs. suture alone, but not vs. stapler alone [76]. CR-POPF rates were not reported. Anastomotic closure of the pancreas with a pancreaticojejunostomy was also compared and showed a benefit over suture closure alone for total POPF. There was no comparison of stapler and anastomotic closure. Thus, overall, stapled closure of the pancreas is not inferior to suture ligation, although it is hard to definitively say if it is superior.

The PANDORINA trial was a randomized trial conducted in the Netherlands and Italy [77]. Patients were randomized to either surgical drain placement after pancreatic resection or no drain placement. Drains were removed on the third post-operative day if the volume of drainage was below 200 mL and if the drain amylase level was less than 3 times the upper limit of normal. Interestingly, patients that did not have a drain placed at the time of surgery had a significantly lower rate of CR-POPF (12% vs. 27%). There were 3 deaths in the group without drains (not statistically significant); however, 2 of these cannot be attributed to the trial interventions, and major morbidity was not significantly different between groups (15% vs. 20%). It is also interesting that in a subgroup analysis of patients deemed high risk for CR-POPF pre-operatively, there is no longer a benefit for drain omission to reduce CR-POPF. Thus, patients at high risk for CR-POPF should be considered a unique cohort, and operative drain placement may still play an important role in their management. The authors hypothesize that drain placement may cause infection of benign intra-abdominal post-operative fluid collections and thus lead to clinically significant complications such as CR-POPF. They therefore advocate no drain placement after distal pancreatectomy, although with a stipulation that results should not be generalized to patients with an ASA score of 4–5 who were excluded from this study and may be prone to more serious complications [77]. These results are further supported by a meta-analysis that reviewed two randomized studies and six comparative studies that all tended to favor drain omission in order to reduce CR-POPF [78]. Again, it is important to consider patient selection, as patients at high risk for CR-POPF may need individualized treatment algorithms.

Multiple studies have also evaluated early post-operative drain removal, both after PD and DP. A meta-analysis reviewed one randomized trial and four non-randomized studies in a subgroup analysis of patients undergoing DP specifically [79]. The definition of early drain removal varied somewhat from study to study and was defined as drain removal sometime between post-operative day 1 and 5. Results showed that CR-POPF was significantly reduced with early drain removal, as were rates of total complications. Unfortunately the randomized trial only included 40 patients total, and thus these conclusions are driven primarily by the non-randomized studies. It is also important to recognize that 2 of the 5 studies in this meta-analysis selected low-risk patients by using a drain amylase cut-off level below 5000 for patient inclusion. Nonetheless, current evidence suggests that drain omission or early drain removal may be reasonable in patients determined to be at low risk for CR-POPF.

### 4.3. Post-Operative Strategies for Reducing CR-POPF

In the immediate post-operative setting, numerous studies have evaluated the use of medications to promote pancreatic healing and reduce the risk for CR-POPF (Table 5). In particular, somatostatin analogues have garnered significant attention as a means to potentially biochemically reduce pancreatic secretions and thus promote healing. Octreotide has been studied since the 1990s, but more recently, there has been increased attention to other agents such as pasireotide [81,82]. A notable randomized trial of 300 patients that underwent pancreatic resection at Memorial Sloan Kettering (MSK) showed significant reduction in a composite endpoint of clinically significant pancreatic leak, fistula, and abscess with the use of pasireotide for 7 days [82]. This trial included 80 patients that underwent DP, and this subgroup also showed a significant reduction in the primary endpoint (7% vs. 23%). The authors also published a follow-up study five years later reporting subsequent outcomes since the adoption of ubiquitous pasireotide use in all pancreatic resections [83]. They show persistently low levels of pancreatic leak/fistula/abscess in the 652 patients treated since the introduction of pasireotide. The composite endpoint rate was 13.3% for both PD and DP combined, significantly lower than their historic rate of 20.9%. Individual rates for PD and DP alone were not reported but appear almost identical, with DP having a higher rate of complication in their prior control cohort. Unfortunately, the favorable results of this study have not been able to be replicated at other institutions, although the studies were not randomized. Two cohort studies were performed at separate institutions and showed no significant reduction in CR-POPF rates compared to their own historic controls [84,85]. This was true for PD and DP. As a result, a meta-analysis evaluating the data of the randomized study and subsequent three cohort studies found no significant reduction in CR-POPF in DP with the use of pasireotide [86]. However, it is important to note that the difference seen in the original RCT was driven by the subgroup of patients with a dilated pancreatic duct and those who underwent distal pancreatectomy. Analysis focused on these subgroups was not performed in the cohort studies, and risk stratification was different. In addition, the sample size of DP in each study is limited. Pasireotide may be a valid consideration and was recommended by an expert panel of surgeons for use in pancreatic resections at high risk for CR-POPF, but convincing evidence validating its efficacy is lacking [87].

Other somatostatin analogues have also been studied. A single-arm study of lanreotide found promising results [88]. In the study, 36 patients underwent DP and were given a single pre-operative dose of lanreotide. The CR-POPF rate was low at 2.7%, indicating that further study in a randomized setting is worthwhile. Lanreotide in particular has the benefit of only requiring one depot administration to last throughout the post-operative period with a long half-life of 23 days [88]. Continuous infusion with somatostatin was studied in a multicenter randomized study from France [89]. Somatostatin has a higher binding affinity to its receptors than octreotide and therefore was hypothesized to potentially have a stronger therapeutic effect. However, the study showed equivalent rates of CR-POPF in the two study arms (17.6% in the somatostatin group vs. 16.5% in the octreotide group). Efficacy of either drug against a placebo control was not performed in this study. Another meta-analysis reviewed various somatostatin analogues in PD and DP [90]. In the DP subgroup, two randomized trials of octreotide were included, as well as the randomized MSK study of pasireotide. While the combination of the three studies showed a significant reduction in CR-POPF with somatostatin analogue administration, this was driven by the pasireotide trial from MSK. The two randomized trials of octreotide did not show a significant reduction in CR-POPF [90]. Overall, the benefits of various somatostatin analogues to reduce CR-POPF remain controversial, and surgeon practices vary widely.

Hydrocortisone has also been evaluated in pancreatic surgery. It is hypothesized to reduce inflammation and edema, thereby promoting pancreatic healing. An initial randomized study from Finland included 31 patients undergoing DP [91]. Patients in the experimental group were treated with 100 mg hydrocortisone IV given 3 times per day for 9 doses total. They showed a significant reduction in CR-POPF compared to placebo (5.9% vs. 42.9%). Of note, all surgeries were performed in an open fashion, and all pancreatic transections were performed with a scalpel with hand-sewn closure of the pancreatic stump. The high rate of CR-POPF in the control group is attributed to only high-risk patients with a soft pancreatic gland being enrolled in the study. A subsequent randomized study compared the efficacy of hydrocortisone with pasireotide [92]. Here, 60 patients that underwent DP were randomized to either 3 days of hydrocortisone or 7 days of pasireotide. While CR-POPF rates were not statistically different between the two groups (20% with hydrocortisone vs. 13% with pasireotide), the rates of total POPF were increased in the hydrocortisone group (67% vs. 37%), and the rates of major complications were increased in the hydrocortisone group (20% vs. 0%). There was no placebo control arm to compare to. Based on this study, hydrocortisone is inferior to pasireotide in DP, but its benefits over placebo remain unclear.

### 4.4. Future Prospects: Pre-Clinical Studies to Reduce CR-POPF

Multiple studies have investigated novel ways to reduce POPF after DP (Table 6). One strategy employed by several groups involves the use of a manufactured sheet, scaffold, or hydrogel that is applied to the cut edge of the pancreas to help the tissue heal. These work both by enhancing wound healing and by reducing the severity of any leak that does develop by trapping caustic enzymes. The earliest such model used sheets of rat myoblast cells that were first grown in vitro and then applied to the cut edge of the pancreas at the time of surgery [93]. Myoblast sheets were chosen because of their regenerative properties in other organs, such as tissue repair after ischemic heart failure. Other groups have instead focused on stem cell sheets. Adipose-derived stem cells (ADSCs) and bone marrow-derived stem cells (BMSCs) were formed into sheets and applied to the cut edges of a rat pancreas [94,95]. ADSCs were chosen because of their abundance and availability for harvest, as well as their regenerative properties as evidenced by studies of ischemia in the colon, liver, and heart [94]. The addition of mannose, a carbohydrate that may support ADSC function, further reduced the levels of amylase and lipase in the rat ascites fluid after POPF creation. BMSCs were used for their ability to differentiate and reduce inflammation. Both ADSCs and BMSCs showed similar success in reducing ascites volume and ascites amylase levels in a rat POPF model [95].

Non-cellular sheets have also been studied in POPF models because of concern that using stem cells in the setting of cancer may compromise oncologic results. Polyglycolic acid (PGA) is a biodegradable polymer commonly used to make certain surgical sutures. This material was manufactured into sheets and tested in a rat model of POPF because of the ability for PGA to act as a scaffold for tissue regeneration [96]. Similarly, polyvinyl alcohol (PVA) is another synthetic polymer that is bio-compatible and was selected for its chemical resistance and utility in wound healing [97]. Another type of hydrogel was developed by He et al. by screening for peptides with affinity towards the pancreatic enzymes trypsin, chymotrypsin, and lipase [98]. This led to the generation of a chiral peptide hydrogel, termed CP-CNDS. This hydrogel was shown to draw in digestive pancreatic enzymes and thus reduces the severity of POPF in a rat model [98].

All of these studies of cell sheets, scaffolds, and hydrogels were performed using rat models of POPF, and they had similar success in showing reduced levels of amylase and lipase in the ascites fluid by the addition of these agents. Inflammation was also reduced, as measured macroscopically by the degree of adhesions and also as measured histologically. The most significant difference seen in the experimental group was perhaps in the study by He et al., which evaluated a peptide hydrogel and showed a complete survival in their experimental animal cohort, as compared to untreated rats that uniformly died [98]. Large animal studies and the development of clinical trials using these approaches are the next steps towards improving CR-POPF rates following DP.

Another approach in pre-clinical models has been the use of injectable drugs to reduce POPF. One study combined three drugs that were administered to rats for 1 week: octreotide, gabexate mesilate, and imipenem/cilastatin [99]. Gabexate mesilate is a protease inhibitor used in Japan to treat pancreatitis by suppressing trypsin activity, and imipenem/cilastatin was added as an antibiotic to reduce infectious complications of POPF. This study showed a reduction in amylase and lipase in ascites fluid, as well as reduced intra-abdominal adhesions and inflammation with the use of these systemic medications in a rat model [99]. A different study instead focused on direct intra-pancreatic injections of penicillin G [100]. This was studied in mice and showed that penicillin G injections can cause reversible fibrosis and hardening of the pancreatic tissue through the TGF-β1 pathway [100]. This was not tested further in a POPF model but may prove beneficial as a pre-operative approach to create a gland that is easier to ligate and seal.

Finally, several studies have looked at novel ligation devices to help mechanically seal the pancreas. One study used a 3D-printed plastic band to encircle and ligate the pancreatic parenchyma just adjacent to the transection line. When arterial blood flow was preserved to the pancreatic stump, there was no post-operative fluid collection seen on CT 1 week after the operation and reduced pancreatic necrosis [101]. Another study fabricated a biodegradable polycaprolactone clip to seal the pancreas prior to transection [102]. This device was also developed to seal the pancreatic duct while avoiding crush injury to the pancreatic parenchyma. Pigs that underwent DP had no ascites or evidence of biochemical or clinical POPF when treated with this new clip [102]. Overall, these various sealants, medications, and surgical devices offer exciting prospects for improving the clinical care of patients undergoing distal pancreatectomy. Follow-up clinical studies will determine if these innovative solutions prove to be effective in practice, and it is noteworthy that many of these techniques could be applied in concert for potentially synergistic effects.

## 5. Conclusions

Despite significant advancements in both surgical and peri-operative care, CR-POPF remains a significant source of morbidity and mortality for patients that undergo DP. While certain risk factors such as obesity, thick pancreatic gland, and absence of diabetes have been well-established as some of the predictors for CR-POPF, it still remains difficult to anticipate exactly which patients will develop this complication. Importantly, cancer patients that undergo DP and suffer from CR-POPF are more likely to have delays in their adjuvant chemotherapy initiation and have reduced PFS and OS. Extensive efforts have been devoted to improving surgical techniques and to identifying ways of reducing CR-POPF. Unfortunately, almost none have had consistent success across multiple trials and different institutions. There is a growing body of evidence that drain omission or early drain removal may be feasible in patients deemed low-risk for CR-POPF. However, there are also exciting new developments with novel hydrogels for enzymatic sequestration and devices for improved pancreatic sealing. Future trials are awaited expectantly to see if patient care can be improved further.

## Figures and Tables

**Table 1:** Risk factors for CR-POPF after DP. The table includes risk factors that have been identified consistently across multiple studies, while those that have conflicting support have been omitted.

Clinical Factor	Association with CR-POPF	Studies
Smoking	Increased risk	[3]
Diabetes mellitus	Decreased risk	[3,5,6]
Open approach	Increased risk	[3]
High BMI	Increased risk	[5,6,7,8]
Young age	Increased risk	[5,7,9]
Pancreatic thickness	Increased risk	[4,5,6,8]
Hypoalbuminemia (<3.5 g/dL)	Increased risk	[7]
Lack of epidural anesthesia	Increased risk	[7]
Neuroendocrine or non-malignant pathology	Increased risk	[7,10]
Splenectomy	Increased risk	[7]
Vascular resection	Increased risk	[7]
Longer operative time	Increased risk	[4,8,9]
High drain fluid amylase on POD1 and POD3	Increased risk	[9]

**Table 2:** Complications associated with and exacerbated by CR-POPF after DP.

Complications	Risk of Complication with CR-POPF	Studies
Intra-abdominal abscess	25–55%	[18,19,20]
Delayed gastric emptying (DGE)	2–24%	[21,22,23]
Hemorrhage	3–15%	[24,25]
Sepsis	2–10%	[26,27]
Wound infection	3–11%	[28,29]
Reoperation	3–6%	[26,30]
Re-admission	11–23%	[31,32,33]
Mortality	0.5–4%	[31,33,34]
Hernia	4–20%	[35,36]

**Table 3:** Clinical studies of pre-operative strategies for CR-POPF prevention/reduction. Primary experimental agents, the study design, and major findings are listed. RCT: randomized controlled trial; OR: odds ratio.

Intervention	Population	Outcome (CR-POPF Reduction)	Conclusion	Reference
Prophylactic PD stenting	Single cohort study, 9 patients, Japan	0% CR-POPF	May offer benefit, needs further validation	[43]
Prophylactic PD stenting	Non-randomized cohort study, 48 patients, Germany	↓POPF: 0% vs. 22%	May offer benefit, needs further validation	[44]
Prophylactic PD stenting	RCT, 58 patients, Sweden	No change in CR-POPF: 42% vs. 22%	No reduction in POPF	[45]
Prophylactic PD stenting (meta-analysis)	Meta-analysis: 1 RCT, 3 non-RCT, 200 patients	↓CR-POPF: 0.45 OR (CI 0.22–0.94)	May offer benefit, but a single RCT conflicts with other results	[48]
Botulinum toxin injection	Non-randomized cohort study, 48 patients, Germany	↓CR-POPF: 0% vs. 33%	May offer benefit, needs further validation	[49]
Botulinum toxin injection	Non-randomized cohort study, 38 patients, Germany	No change in CR-POPF: 32% vs. 42%	No clear benefit, needs further validation	[50]

**Table 4:** Clinical studies of intra-operative strategies for CR-POPF prevention/reduction. The primary experimental agent, the study design, and major findings are listed. RCT: randomized controlled trial; OR: odds ratio; RR: risk ratio.

Intervention	Population	Outcome (CR-POPF Reduction)	Conclusion	Reference
Fibrin Sealant Patch (TachoSil)	Multicenter RCT, 275 patients, 19 centers, Italy	No significant ↓ in CR-POPF: 8% vs. 14% without	No benefit, but ↓ drain amylase levels on POD1	[52]
Fibrin Sealant Patch (TachoSil) (FIABLE study)	Multicenter RCT, 270 patients, 45 centers, France	No significant ↓ in CR-POPF: 31% vs. 24% without	No benefit	[53]
Fibrin Sealant Patch (TachoSil)	Multicenter RCT, 101 patients, 5 centers, South Korea	No significant ↓ in CR-POPF: 23% vs. 28% without	No benefit	[54]
Fibrin Sealant Patch (TachoSil) (meta-analysis)	Meta-analysis: 2 RCTs, 2 non-RCTs, 738 patients	No significant ↓ in CR-POPF: 17.8% vs. 18.3% without	No benefit	[55]
Fibrin Sealant Patch (TachoSil) (CPR trial)	Multicenter RCT, 247 patients, 7 centers, The Netherlands	No significant ↓ in CR-POPF: 20% vs. 24% without	No benefit	[56]
Reinforced stapler (PGA or biologic)	RCT, 100 patients, USA	↓CR-POPF: 1.9% vs. 20%	Supports mesh-reinforced stapler	[57]
PGA mesh wrap	Multicenter RCT, 97 patients, 5 centers, South Korea	↓CR-POPF: 11.4% vs. 28.3%	Supports PGA mesh wrap	[58]
Reinforced stapler (PGA)	Multicenter RCT, 119 patients, 9 centers, Japan	No significant ↓ in CR-POPF: 16% vs. 27% without	May have been underpowered; benefit in pancreas <14 mm thick	[59]
Reinforced stapler (biologic)	Multicenter RCT, 106 patients, 4 centers, Sweden	No significant ↓ in CR-POPF: 11% vs. 16% without	No benefit with biologic reinforcement	[60]
Reinforced stapler (PGA) (REPLAY study)	Multicenter RCT, 199 patients, 7 centers, France	No significant ↓ in CR-POPF: 14% vs. 11% without	No benefit with PGA reinforcement	[61]
PGA mesh (meta-analysis)	Meta-analysis: 3 RCTs, 3 non-RCTs, 615 patients	↓CR-POPF: RR 0.31 (CI 0.21–0.46)	Benefit of PGA mesh reinforcement	[62]
Reinforced stapler (meta-analysis)	Meta-analysis: 2 RCTs, 5 non-RCTs, 553 patients	↓CR-POPF: OR 0.33 (CI 0.19–0.57)	Benefit to reinforced stapler, but heterogeneity between studies	[63]
Reinforced Stapler (meta-analysis)	Meta-analysis: 3 RCTs, 5 non-RCTs, 743 patients	No significant ↓ in CR-POPF: OR 0.79 (CI 0.47–1.35)	Reinforced stapler not effective per RCTs	[64]
Teres ligament patch (DISCOVER trial)	RCT, 152 patients, Germany	No significant ↓ in CR-POPF: 22% vs. 33% without	Fewer reoperations/re-admissions with patch	[65]
Patch coverage (autologous) (meta-analysis)	Meta-analysis: 5 RCTs, 6 non-RCTs, 1359 patients	↓CR-POPF: RR 0.50 (CI 0.32–0.78)	Autologous patches reduce CR-POPF but not fibrin patches	[66]
Cyanoacrylate glue	Pilot study, 15 patients (terminated early), Germany	CR-POPF: 33.3%	Feasible, but no obvious benefit in small sample size	[67]
Flowable hemostatic matrix vs. thrombin patch	RCT, 25 patients, South Korea	↓CR-POPF: 8.3% vs. 46.2%	Flowable hemostatic matrix outperforms thrombin-coated patch	[68]
Hemopatch	Multicenter RCT, 315 patients, 17 centers, Europe	No significant ↓ in CR-POPF: 16% vs. 23% without	No benefit for CR-POPF, but fewer complications overall	[69]
Hemopatch (PATCH-DP trial)	Multicenter single-arm trial, 52 patients, 7 centers, Canada	No significant ↓ in CR-POPF: 25% (vs. historical baseline 20%)	No benefit for CR-POPF, but limited study	[70]
Actiseal patch (SHIELDS)	Multicenter single-arm trial, 40 patients, 8 centers, Europe	CR-POPF: 17.5%	Feasible; RCT needed	[71]
Non-autologous reinforcement (meta-analysis)	Meta-analysis: 9 RCTs, 1497 patients	↓CR-POPF: RR 0.677 (CI 0.479–0.956)	Non-TachoSil reinforcement is effective	[72]
Prolonged stapler compression	Retrospective, 42 patients, Japan	↓CR-POPF: 5.9% vs. 32% without	Slow compression is better than no compression	[73]
No stapler compression	Retrospective, 59 patients, Japan	↓ CR-POPF: 7.5% vs. 31.6% without	No compression is better than slow compression	[74]
Stapler and suture vs. stapler alone	Cohort study, 22 patients, Japan	↓ CR-POPF: 0% vs. 33% without	Combined stapler/suturing seems to reduce CR-POPF	[75]
Stapler vs. suture vs. anastomosis vs. combined techniques (meta-analysis)	Meta-analysis: 2 RCTs, 35 non-RCTs, 5252 patients	No significant ↓ in CR-POPF: 14.2% stapler group vs. 17.4% suture group	POPF was reduced with a stapler vs. suture, but CR-POPF was equivalent with both	[76]
No drain vs. drain (PANDORINA trial)	Multicenter RCT, 282 patients, 12 centers, Europe	↓ CR-POPF: 12% without vs. 27% with drain	No drain improves CR-POPF rate	[77]
No drain vs. drain (meta-analysis)	Meta-analysis: 2 RCTs, 6 non-RCTs, 3610 patients	↓ CR-POPF: OR 0.38 (CI 0.25–0.56)	No drain improves CR-POPF rate	[78]
Early drain removal (meta-analysis)	Meta-analysis: 1 RCT, 4 non-RCTs, 5343 patients	↓ CR-POPF: RR 0.17 (CI 0.13–0.24)	Early removal improves the CR-POPF rate (based on non-RCTs)	[79]

**Table 5:** Clinical studies of post-operative strategies for CR-POPF prevention/reduction. Primary experimental agents, the study design, and major findings are listed. RCT: randomized controlled trial; OR: odds ratio.

Intervention	Population	Outcome (CR-POPF Reduction)	Conclusion	Reference
Pasireotide	RCT, 80 DP patients, USA	↓ CR-POPF: 7% vs. 23% without	Benefit to pasireotide	[82]
Pasireotide	Cohort study, 29 DP patients, USA	No significant ↓ in CR-POPF: 27% vs. 11% without	No benefit	[84]
Pasireotide	Cohort study, 81 DP patients, USA	No significant ↓ in CR-POPF: 15% vs. 21% without	No benefit	[85]
Pasireotide (meta-analysis)	Meta-analysis: 1 RCT, 3 non-RCTs, 401 patients	No significant ↓ in CR-POPF: OR 0.70 (CI 0.30–1.63)	No benefit	[86]
Lanreotide	Single cohort study, 36 DP patients, USA	2.7% CR-POPF	May offer benefit, needs further validation	[88]
Somatostatin vs. octreotide (PREFIPS Trial)	Multicenter RCT, 170 DP patients, 15 centers, France	No significant ↓ in CR-POPF: 18% vs. 17%	No benefit to somatostatin over octreotide	[89]
Somatostatin analogues (meta-analysis)	Meta-analysis: 3 RCTs, 209 patients	↓ CR-POPF: OR 0.41 (CI 0.18–0.91)	Benefit to analogues (driven by single RCT), but not octreotide	[90]
Hydro-cortisone	RCT, 31 patients, Finland	↓ CR-POPF: 6% vs. 43% without	Strong promising effect	[91]
Hydro-cortisone vs. pasireotide	RCT, 60 patients, Finland	No significant ↓ in CR-POPF: 20% vs. 13%	No difference in CR-POPF, but more POPF and complications with hydrocortisone	[92]

**Table 6:** Pre-clinical studies of CR-POPF prevention/reduction. The primary experimental agent, the animal model employed in the study, and major findings are listed.

Intervention	Animal Model	Outcome	Conclusion	Reference
Tissue-engineered myoblast sheet patches	Rat splenic duct transection model	↓ ascitic amylase and lipase; ↓ adhesions and inflammation at pancreatic stump	Myoblast sheets may prevent POPF and promote healing	[93]
Mannose-enhanced ADSC sheets	Rat splenic duct transection model	↓ascitic amylase and lipase; ↑ FGF2; improved healing	Mannose-enhanced sheets may reduce POPF	[94]
Mesenchymal stem cell (MSC) sheets from adipose (rADSC) and bone marrow (rBMSC)	Rat DP/splenectomy model	↓ ascites volume; ↓ inflammation; ↓ amylase levels; improved histology	Promising biomaterial approach; enhances healing	[95]
PGA fabric scaffold for pancreatic stump	Rat ventral pancreas cauterization model	↓ peritonitis; ↑ survival; effective barrier with ↑ fibroblast infiltration	PGA scaffold is effective in preventing POPF in rats	[96]
Polyvinyl alcohol (PVA) hydrogel sheet	Rat splenic duct transection model	↓ ascitic amylase and lipase; ↓ saponification; ↓ inflammation	PVA hydrogel may be superior to PGA felt; it is a promising biomaterial	[97]
D-Peptide hydrogel (CP-CNDS) for enzyme trapping	Rat common pancreatic duct transection model	↓ ascitic amylase and lipase; 100% survival vs. 100% 5-day mortality	D-Peptide hydrogel is promising for POPF prevention and enzyme sequestration	[98]
Triple-drug therapy (octreotide + gabexate mesilate + imipenem/cilastatin)	Rat splenic duct transection model	↓ ascitic amylase and lipase; ↓ intra-abdominal adhesions; ↓ inflammation	Suggested efficacy in reducing POPF in rats	[99]
Penicillin G injection for fibrosis	BALB/c mouse model	↑ fibrosis via TGF-β1; ↑ hardness; reversible effects	Penicillin G induces fibrosis and may prevent POPF	[100]
Pancreas ligation band	Porcine distal pancreatectomy model	No POPF in animals with maintained arterial flow; necrosis rates 24–31% (ligated with flow) vs. 83% (without flow)	Pancreas ligation band may reduce POPF by atraumatic ligation, preserving blood flow and reducing necrosis	[101]
Bioabsorbable pancreatic clip (BioPaC device)	Porcine distal pancreatectomy model	BioPaC group: 0% POPF; linear stapler group: 1/2 Grade C POPF; better duct closure	BioPaC may prevent POPF by avoiding compression injury; it is a promising device for DP	[102]

## Abbreviations

The following abbreviations are used in this manuscript:

DP	Distal pancreatectomy
CR-POPF	Clinically relevant post-operative pancreatic fistula
PD	Pancreaticoduodenectomy
